# Optimizing Antioxidant Potential: Factorial Design-Based Formulation of Fucoidan and Gallic Acid-Conjugated Dextran Blends

**DOI:** 10.3390/md22090417

**Published:** 2024-09-13

**Authors:** Cynthia Haynara Ferreira Silva, Maylla Maria Correia Leite Silva, Weslley Souza Paiva, Mayara Jane Campos de Medeiros, Moacir Fernandes Queiroz, Luciana Duarte Martins Matta, Everaldo Silvino dos Santos, Hugo Alexandre Oliveira Rocha

**Affiliations:** 1 Departamento de Bioquimica, Programa de Pós-Graduação em Bioquímica e Biologia Molecular—PPgBBM, Centro de Biociências, Universidade Federal do Rio Grande do Norte—UFRN, Natal 59078-970, Brazil; cynthia.haynara.016@ufrn.edu.br (C.H.F.S.); maylla.correia.104@ufrn.edu.br (M.M.C.L.S.); weslley.paiva.093@ufrn.edu.br (W.S.P.); moacirfqn@gmail.com (M.F.Q.); luciana.matta@ufrn.br (L.D.M.M.); 2 Laboratório de Química de Coordenação e Polímeros (LQCPol), Instituto de Química, Universidade Federal do Rio Grande do Norte—UFRN, Natal 59078-970, Brazil; 3 Laboratório de Engenharia Bioquímica, Departamento de Engenharia Química, Universidade Federal do Rio Grande do Norte—UFRN, Natal 59078-970, Brazil; everaldo.eq@gmail.com

**Keywords:** brown seaweed, fucan, *Spatoglossum schröederi*, oxidative stress, sulfated polysaccharide

## Abstract

The role of oxidative stress in health and homeostasis has generated interest in the scientific community due to its association with cardiovascular and neurodegenerative diseases, cancer, and other diseases. Therefore, extensive research seeks to identify new exogenous antioxidant compounds for supplementation. Polysaccharides are recognized for their antioxidant properties. However, polysaccharide chemical modifications are often necessary to enhance these properties. Therefore, dextran was conjugated with gallic acid (Dex-Gal) and later combined with fucoidan A (FucA) to formulate blends aimed at achieving superior antioxidant activity compared to individual polysaccharides. A factorial design was employed to combine FucA and Dex-Gal in different proportions, resulting in five blends (BLD1, BLD2, BLD3, BLD4, and BLD5). An analysis of surface graphs from in vitro antioxidant tests, including total antioxidant capacity (TAC), reducing power, and hydroxyl radical scavenging, guided the selection of BLD4 as the optimal formulation. Tests on 3T3 fibroblasts under various conditions of oxidative stress induced by hydrogen peroxide revealed that BLD4 provided enhanced protection compared to its isolated components. The BLD4 formulation, resulting from the combination of Dex-Gal and FucA, showed promise as an antioxidant strategy, outperforming its individual components and suggesting its potential as a supplement to mitigate oxidative stress in adverse health conditions.

## 1. Introduction

Cells rely on cellular processes involving redox reactions to maintain their basic functions and carry out essential tasks like cell proliferation and differentiation. These processes operate within a dynamic equilibrium, interacting with regulatory components. However, when there is an imbalance between pro-oxidant and antioxidant agents, there is an upsurge in reactive species production, including reactive oxygen species (ROS), leading to a condition known as oxidative stress [1,2].

This situation can inflict damage on cellular components, jeopardizing cell integrity and proper function. Oxidative stress can act as a triggering factor for various degenerative processes, such as cardiovascular diseases, neurodegenerative disorders, and cancer. This is attributable to the detrimental impact of oxidative stress on cellular function, subsequently affecting tissues and organs and contributing to the onset of these pathological conditions [3,4].

Antioxidant agents can originate from enzymatic or non-enzymatic sources. Non-enzymatic antioxidants, in turn, can stem from either endogenous or exogenous sources [5]. Antioxidant agents from exogenous sources can be obtained through food or supplementation and may help in cases of the dysregulation of endogenous antioxidant sources [6].

An ideal exogenous antioxidant would be able to effectively neutralize a wide range of reactive species. It would be resistant to chemical degradation, maintaining its antioxidant activity over time. Additionally, it would be safe for human and animal consumption, exhibit bioavailability, function effectively in different environments, including both water and lipid-based environments, and be effective at various pH levels. Moreover, it would be cost-effective to produce and, therefore, accessible to a wide range of people [7].

It is important to recognize that no single antioxidant compound encompasses all beneficial properties. Therefore, researchers are continually exploring new antioxidant compounds that offer potential health benefits and can be used in combination with others [8]. 

Polysaccharides are among the natural molecules evaluated for their antioxidant properties [9]. Within this realm, sulfated polysaccharides (PSs) derived from marine algae have garnered significant attention [10,11,12]. The sulfate groups present in these compounds confer a negative charge to the polysaccharides, thereby facilitating antioxidant activity through various mechanisms. However, it is not just the presence of these groups that matters; the quantity and distribution pattern of sulfate groups throughout the PS molecule also contribute to enhancing its antioxidant activity [13,14].

The brown alga *Spatoglossum schröederi*, abundant along the Atlantic coast of South and Central America, synthesizes three sulfated polysaccharides (PSs) known as fucoidans A, B, and C [15,16]. Among these, fucoidan A (FucA), whose chemical structure has already been proposed [17], stands as the most extensively studied. Notably, FucA exhibits non-cytotoxic, non-genotoxic, and non-mutagenic characteristics [18], alongside antithrombotic activity [15]. Moreover, it has been employed in the production of nanogels targeting tumor cells [19] and silver nanoparticles with anti-trypanosome properties [20]. Recent findings highlight FucA’s ability to shield cells from the harmful effects of genotoxic agents [21] and its anti-inflammatory potential [22]. Both activities are related to the antioxidant activity of FucA [21].

Sulfated polysaccharides, such as FucA, have been highlighted as antioxidant agents in various studies [10,23]. In the specific case of FucA, it is worth noting that it is formed by the main chain of glucuronic acid with sulfated L-fucose substitutions. The antioxidant activity of polysaccharides is occasionally attributed to the presence of proteins and phenolic compounds that may contaminate the sample. Therefore, Rodrigues-Souza et al. [21] not only demonstrated that FucA exhibited antioxidant activity but also confirmed that this polysaccharide was free from such contaminants. Therefore, the presence of sulfate and carboxyl groups is considered responsible for the ability of fucoidans to participate in redox processes and act as antioxidant agents [24], which justifies the antioxidant action of FucA.

Some neutral polysaccharides have been identified as agents with low antioxidant activity, such as dextran obtained from *Leuconostoc mesenteroides*. However, after modification with gallic acid (GA), this dextran became a potent antioxidant agent due to the action of GA [25]. The electron donation efficiency of GA depends on its steric freedom, which in turn depends on its substituent [26]. Curcio et al. [27] suggested that GA binds to polysaccharides through its carboxyl group, forming an ester with the polysaccharide at the end of the process. This provides greater stability to GA, allowing its hydroxyl group in the para position to act as a potent reducing agent. The same occurs when GA is conjugated with dextran, making the Dex-GA compound a better antioxidant agent than either dextran or GA alone [25].

One strategy to enhance the antioxidant activity of compounds, including polysaccharides, involves formulating blends. This approach aims to combine two or more components to enhance functions that were previously unattainable when using them in isolation [28,29]. For example, some antioxidants neutralize free radicals directly, while others chelate pro-oxidant metals or modulate antioxidant biochemical pathways. Combining antioxidants in a blend can provide more comprehensive protection against oxidative damage. Additionally, this combination can enhance the stability and bioavailability of the compounds, as certain antioxidants may protect others from degradation or improve their absorption in the body [30,31]. For instance, recent research demonstrated that a blend containing agaran, a sulfated polysaccharide from red seaweed, and chromium picolinate exhibited superior antioxidant activity both in vitro and in vivo compared to these compounds in isolation [32].

Mixtures containing FucA or Dex-GA have not yet been developed. In previous studies, FucA was shown to scavenge OH radicals [25], whereas Dex-GA did not exhibit this activity. Additionally, FucA demonstrated greater activity than Dex-GA in the total antioxidant capacity (TAC) test. Conversely, Dex-GA exhibited higher activity than FucA in the reducing power test [25,28]. Therefore, these two polysaccharides possess complementary antioxidant activities. To validate this, the aim of this study was to produce blends containing FucA and Dex-GA with enhanced activity compared to these polysaccharides in isolation.

## 2. Results

### 2.1. Formulation of Polysaccharide Blends Incorporating FucA and Dex-Gal

#### Conjugation of Gallic Acid with Dextran

The chemical modification of dextran was conducted following the redox methodology previously employed by Queiroz et al. [25] to incorporate gallic acid molecules into the dextran framework. The redox approach establishes an oxidative–reductive milieu utilizing hydrogen peroxide (H_2_O_2_) and ascorbic acid, thereby engendering reactive oxygen species (ROS). These ROS initiate attacks on specific sites within the molecular structure, inducing the formation of regions harboring unpaired electrons that act as anchoring sites for gallic acid via covalent bonding [33,34]. This chemical modification strategy is widely adopted to enhance the antioxidant prowess of molecules, given the robust antioxidative properties of gallic acid. Furthermore, it is esteemed for its environmental compatibility, characterized by the absence of reagents detrimental to the ecosystem [25].

**Figure 1:**
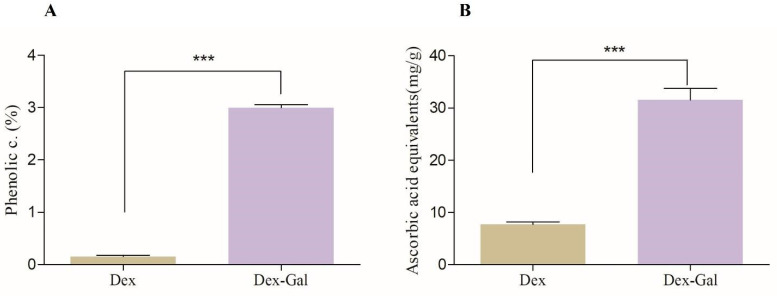
Quantification of phenolic compounds (**A**) and total antioxidant capacity (**B**) of dextran (dex) and modified dextran (Dex-Gal) samples in percentage. Asterisks indicate statistical differences between samples analyzed (*** *p* < 0.0005).

Therefore, dextran (15 kDa) was subjected to modification through conjugation with gallic acid, as outlined in the methodology. Figure 1A,B depict the outcomes of this chemical alteration.

The quantification of phenolic compounds aimed to assess the presence of gallic acid (GA) following the chemical modification process. As depicted in Figure 1A, unmodified dextran (Dex) shows values close to zero, whereas modified dextran (Dex-Gal) contains 3% phenolic compounds.

In Figure 1B, another significant piece of evidence supporting the chemical modification of dextran is presented. The total antioxidant capacity test was conducted by comparing the activity of dextran and Dex-Gal. It is evident that Dex-Gal exhibits a 3-fold increase in antioxidant activity compared to unmodified Dex. These data suggest that the binding of gallic acid (GA) through the redox reaction did not impede the electron-donating activity of the GA hydroxyl groups. Consequently, it led to the formation of a molecule with enhanced antioxidant capacity compared to unmodified dextran (Dex).

These findings align with those reported by Queiroz et al. [25] and other studies investigating chemical modifications involving various polysaccharides. Fernandes-Negreiros et al. [35] observed a gallic acid (GA) percentage of approximately 1.5% upon modifying laminarin with a molecular weight of approximately 12.6 kDa. Similarly, Paiva et al. [36] reported analogous values, approximately 4% GA, when modifying chitosan, while Melo et al. [37] noted a GA content of 3.7% upon modifying FucA.

It is well documented in the literature that molecular weight plays a pivotal role in determining the quantity of gallic acid molecules incorporated. Given that the dextran utilized in this study possesses a molecular weight of 15 kDa, it is plausible to infer that its relatively smaller size may have influenced the percentage of GA insertion. Queiroz et al. [25] proposed that steric hindrance affects the insertion of GA molecules, suggesting that larger molecules offer more potential insertion sites for GA with less intermolecular interaction. Consequently, smaller molecules present fewer insertion points and a higher likelihood of intermolecular interaction, thereby impeding the incorporation of GA into the modified compound.

**Figure 2:**
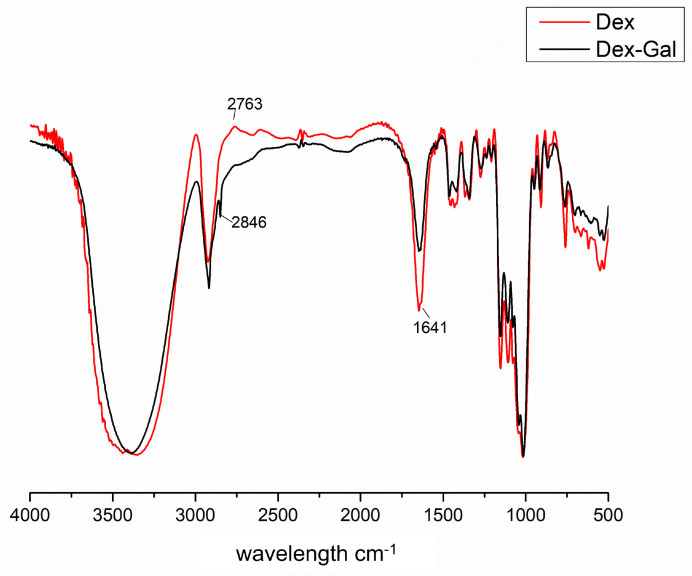
Overlay of FT-IR spectra of 15 kDa dextran (Dex) (red) and modified dextran (Dex-Gal) (black).

In addition to assessing the gallic acid percentage as an indicator of the success of dextran chemical modification, the Fourier-transform infrared (FT-IR) spectra of Dex and Dex-Gal can provide insights into the incorporation of gallic acid. Therefore, the two polysaccharides were also subjected to FT-IR analysis to confirm the chemical modification. Figure 2 shows the FT-IR spectrum of Dex (red) and Dex-Gal (in black), highlighting the main bands found. 

The main bands that are characteristic of polysaccharides and that were found in both spectra are listed in Table 1.

Upon the examination of both spectra and Table 1, notable similarities emerge, suggesting the presence of characteristic signals of gallic acid and preservation of the dextran structure post-modification. Specifically, prominent features include the OH vibration band around 3400 cm^−1^, the glycosidic bond (C-O-C) vibration around 1016 cm^−1^, and the representation of the α-D-glucose configuration at 866–869 cm^−1^ [38]. These spectral resemblances corroborate the successful incorporation of gallic acid into the dextran matrix while retaining its fundamental structural integrity.

However, it is noteworthy that certain bands appear exclusively in the Dex-Gal spectrum, indicative of gallic acid (GA) binding within the molecule. The band observed at 1543 cm^−1^ is characteristic of the vibration of the aromatic ring, a structural feature inherent to gallic acid. Additionally, the band appearing at 1743 cm^−1^ signifies the C=O bond of the ester group, which arises from the covalent linkage between the carboxyl group of gallic acid and the hydroxyl group of the sugar residues within dextran. These spectral signatures are in accordance with observations by Queiroz et al. [25] and validate the connection between gallic acid and dextran, thereby forming Dex-Gal.

With the confirmation of successful chemical modification and the observed increase in the antioxidant activity of Dex-Gal, this molecule presents a promising candidate for formulating blends containing fucoidan (FucA) with the intention of enhancing a compound with heightened antioxidant efficacy.

### 2.2. An Assessment of the Influence of the Amount of FucA and Dex-Gal on the Antioxidant Activity of the Blends Obtained

Factorial planning, an experimental technique grounded in statistical principles, serves as a valuable resource for evaluating combinations of various factors to optimize outcomes with minimal effort [39]. By leveraging this tool, researchers can systematically assess different experimental conditions in terms of their impact on specific response variables, thereby obtaining precise insights into the influence of the factors involved while conducting fewer experiments [40]. 

The utilization of factorial planning in blending compositions containing FucA and Dex-Gal aims to streamline the identification of the most promising proportions with respect to antioxidant activity enhancement. This entails determining the optimal ratio of each component to maximize antioxidant efficacy. The implemented planning strategy facilitated the creation of a matrix comprising five formulations (blends), each representing distinct combinations of FucA and Dex-Gal proportions, for the evaluation of key variables such as total antioxidant capacity, hydroxyl radical scavenging, and an assessment of reducing power.

Here, FucA and Dex-Gal served as the two factors analyzed, while antioxidant activities were designated as the response variables. Five blends, denoted as BLD1, BLD2, BLD3, BLD4, and BLD5, were formulated with varying combinations of FucA and Dex-Gal proportions, as delineated in the methodology, to assess the response variables: total antioxidant capacity (TAC), reducing power, and hydroxyl radical scavenging.

Table 2 presents a summary of the results pertaining to the response variables, i.e., the antioxidant activities, for the five formulated blends.

**Figure 3:**
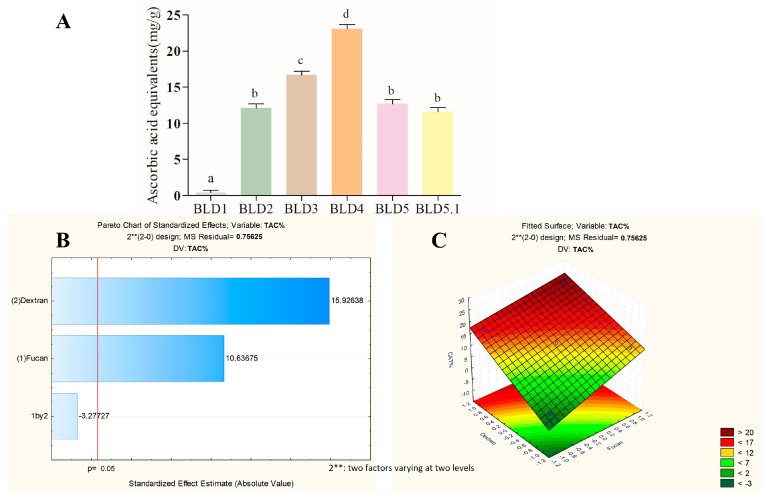
An analysis of the response variable of the total antioxidant capacity of the blends prepared after the factorial design. (**A**) Total antioxidant capacity values; (**B**) Pareto diagram at a significance level of 95%; (**C**) response surface graph. The letters a–d indicate statistical differences between the analyzed samples (*p* < 0.05).

**Figure 4:**
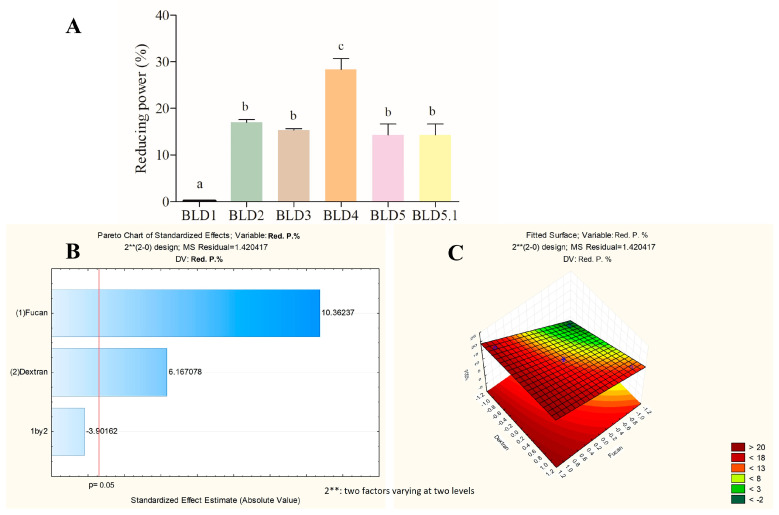
An analysis of the reducing power activity response variable of the blends prepared by factorial planning. (**A**) Reducing power values (%). (**B**) Pareto diagram at a significance level of 95%. (**C**) Response surface graph. The letters a–c indicate statistical differences between the analyzed samples (*p* < 0.05).

**Figure 5:**
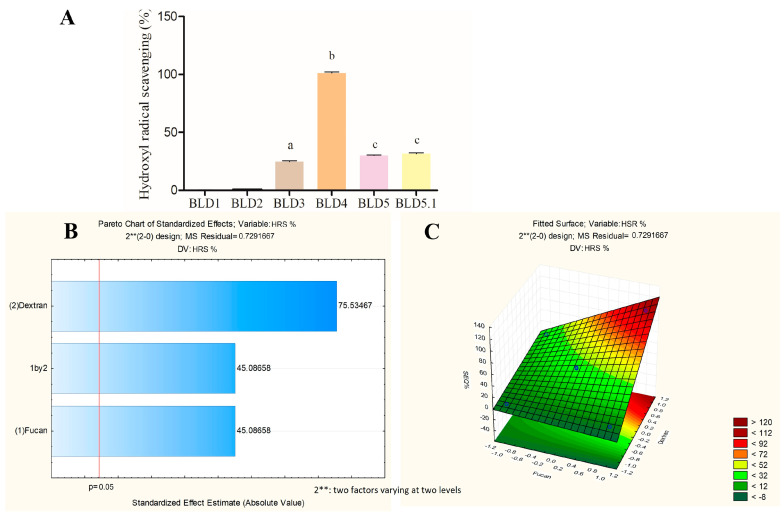
An analysis of the response variable of the hydroxyl radical scavenging activity of the blends prepared by the factorial design. (**A**) The values of hydroxyl radical scavenging activity (%). (**B**) A Pareto diagram at a significance level of 95%. (**C**) Response surface graph. The letters a–c indicate statistical differences between the analyzed samples (*p* < 0.05).

Figure 3, Figure 4 and Figure 5 present the data obtained from quantitative tests assessing the antioxidant potential of the blends formulated in the experimental design, along with the analysis of factorial planning utilizing the Pareto diagram and response surface graph.

The Pareto diagram scrutinizes the hierarchical influence of the analyzed factors on the response variable, illustrated graphically. It enables the evaluation of factors that significantly impact the analyzed response. The index depicted by the Pareto diagram may be positive, signifying that the factor positively affects the response, or negative, indicating a detrimental influence on the evaluated response [41].

The response surface graph illustrates the optimal conditions tested, where the evaluated response demonstrates its peak performance. This graph aids in determining the optimal formulation of the blend, which maximizes the activity under evaluation.

In Figure 3A, the total antioxidant capacity of the blends prepared in the factorial design ranged from 0 to 23.1 equivalents of ascorbic acid. These values were subsequently analyzed in the Pareto diagram to ascertain the influence of FucA and Dex-Gal (Figure 3B). The positive indices indicate that both factors positively affect the total antioxidant capacity, with a statistical significance level of 95%. Notably, Dex-Gal demonstrates a greater influence compared to FucA.

The response surface graph (Figure 3C) delineates the region where the optimization of the total antioxidant capacity response variable occurs. It suggests that the optimal combination for this activity lies within the region where both FucA and Dex-Gal exhibit positive effects. Accordingly, the BLD4 sample, with a coding of (+1, +1), is identified as possessing the optimal blend composition for this activity.

In Figure 4A, the reducing power activity of the samples prepared in the factorial design ranged from 0 to 19.7%. These values were then subjected to analysis in the Pareto diagram to ascertain the influence of FucA and Dex-Gal (Figure 4B). The positive indices suggest that both factors positively affect the reducing power activity, with a statistical significance level of 95%. Notably, FucA demonstrates a greater influence compared to Dex-Gal.

The response surface graph (Figure 4C) delineates the region where the optimization of the reducing power activity occurs. It suggests that the optimal combination for this activity lies within the region where both FucA and Dex-Gal exhibit positive effects. Accordingly, the BLD4 sample, coded as (+1, +1), is identified as possessing the optimal blend composition for this activity.

In Figure 5A, the hydroxyl radical scavenging activity of the samples prepared in the factorial planning ranged from 0 to 103%. These values underwent analysis in the Pareto diagram to determine the influence of FucA and Dex-Gal (Figure 5B). The positive indices indicate that both factors positively affect the radical scavenging activity, with a statistical significance level of 95%. Notably, Dex-Gal demonstrates a greater influence compared to FucA.

The response surface graph (Figure 5C) delineates the region where the optimization of the total antioxidant capacity response variable occurs. It suggests that the optimal combination for this activity lies within the region where both FucA and Dex-Gal exhibit positive effects. Accordingly, the BLD4 sample, coded as (+1, +1), is identified as possessing the optimal blend composition for this activity.

**Figure 6:**
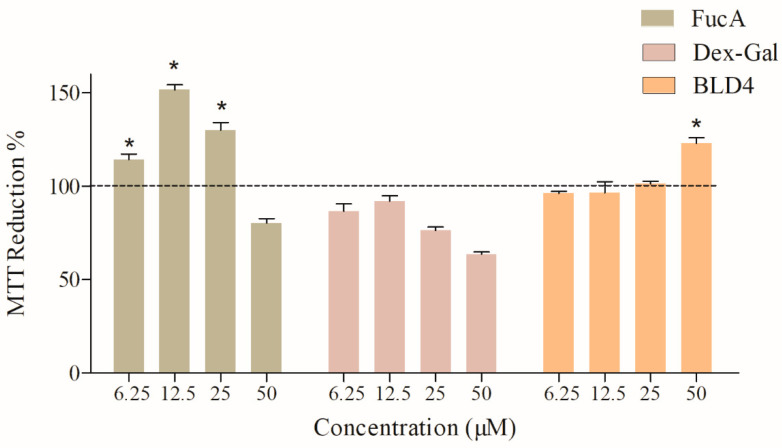
The reduction of MTT to formazan by 3T3 cells in the presence of FucA, Dex-Gal, and BLD4 at concentrations of 6.25 to 50 μM. The symbol * indicates statistical difference between the samples and the control (*p* < 0.05).

In Figure 3B, Figure 4B, and Figure 5B, it is evident from the Pareto diagram that FucA and Dex-Gal exert differing influences on each analyzed antioxidant activity. However, it is crucial to emphasize that the surface graphs depicting the tested activities (Figure 3C, Figure 4C, and Figure 6C) reveal a direct relationship between both FucA and Dex-Gal in optimizing the antioxidant activities under examination. This suggests that the combined effect of both factors supersedes the individual contributions. Specifically, the combination of factors in the BLD4 blend demonstrates an optimal enhancement in the variables.

The formulation of blends represents a strategic approach to amalgamating the benefits of two or more components, surpassing their individual actions [28,29]. Since FucA and Dex are two structurally distinct polysaccharides, it is understandable that they would exert differing influences on the antioxidant activities of the blends obtained in this study. However, the optimization mechanism of antioxidant activity through the blend formulation of FucA and Dex-Gal remains unclear.

Nevertheless, an examination of the structures of these two polysaccharides offers some insight into the structure–antioxidant activity relationship. It is well documented that the biological activities of polysaccharides are closely tied to their structures [42]. Factors such as chain size, monosaccharide composition, branches, quantity, type of substituents, and their distribution within the polysaccharide chain can influence the observed antioxidant activity [11,43]. Therefore, these characteristics could account for the antioxidant activity of the blends. However, they fail to elucidate the differences in antioxidant activities between different blends.

In addition to the presence of functional groups (OH, C-H, SO_4_, GA) being crucial for conferring antioxidant activity upon a polysaccharide, these groups must also be accessible in solution to act as antioxidant agents. In essence, the conformation that the polysaccharide assumes in solution is a predominant factor in its ability to exhibit antioxidant activity [42]. In addition, the effectiveness of antioxidant compounds may also be related to the concentration and presence of synergistic substances [44].

All the blends evaluated in this study consist of the same polysaccharides, thus sharing identical functional groups. Consequently, the primary point of differentiation between them lies in the proportion of FucA and Dex-Gal used in each composition. Hence, it is postulated that these two polysaccharides, when interacting, alter each other’s conformations, with this effect being directly influenced by the polysaccharide concentration. This phenomenon justifies the disparity in antioxidant activity observed among the blends. Future studies are warranted to substantiate this proposition.

Blends containing polysaccharides and other antioxidant agents have been studied for some time. For instance, a blend of β-glucan extracted from oats and polyphenols from tea (*Camellia sinensis*) was found to be significantly more effective as an antioxidant in the DPPH and hydroxyl radical scavenging tests compared to its isolated components [45].

More recently, Zhou et al. [46] created a blend (TPS-Fe(III)) using polysaccharides from Qingzhuan Dark Tea and iron(III) salts. They observed an increase in antioxidant potential relative to the native polysaccharide. As the concentration increased, the DPPH free radical scavenging rate improved correspondingly. At a concentration of 1.0 mg/mL, the DPPH radical scavenging activities of the native polysaccharide and the TPS-Fe(III) complex were 31.56% and 51.55%, respectively. Additionally, the antioxidant activity against hydroxyl radicals also improved significantly, with the polysaccharide–iron sulfate complex showing twice the activity compared to the native polysaccharide—7.67% for the native polysaccharide versus 15.04% for the complex at a concentration of 1.0 mg/mL.

These examples highlight that, as observed in our study, the incorporation of polysaccharides can enhance the antioxidant activity of blends compared to their isolated components. However, blends composed solely of polysaccharides are less commonly studied. One notable example found was a study where xylan oligosaccharides, when combined in a blend, exhibited antioxidant activity in the DPPH test, while the isolated oligosaccharides did not show such activity [47].

Therefore, based on the data gathered thus far, the BLD4 blend was selected to proceed with further investigations.

### 2.3. Assessment of BLD4 Cytotoxicity in 3T3 Cells

To assess the impact of the samples on 3T3 cells, the colorimetric MTT reduction assay was conducted to form formazan. Figure 6 illustrates the obtained results. Across all tested concentrations (except for Dex-Gal 50 µM), all samples induced MTT reduction percentages equal to or exceeding 80%. Notably, FucA and BLD4 exhibited percentages close to 100%. These findings suggest that the samples demonstrate negligible cytotoxicity under most of the evaluated conditions.

### 2.4. Effect of BLD4 on H_2_O_2_-Induced Oxidative Stress in 3T3 Cells

After oxidative stress-induced damage, it is reasonable to expect that such stress may recur. In this case, an antioxidant that acts in conjunction with the stressor, inhibiting it, would be beneficial. This also highlights the importance of using antioxidants that can act preventively against oxidative stress-induced damage.

**Figure 7:**
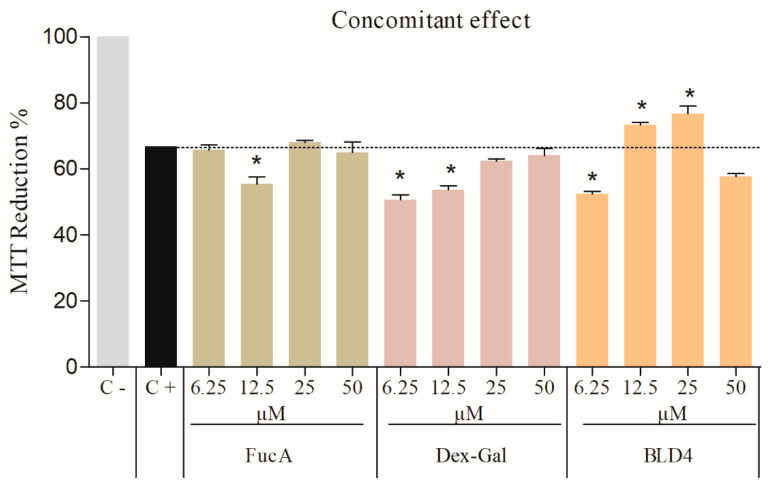
Oxidative stress induced by hydrogen peroxide—concomitant effect. The reduction of MTT to formazan by 3T3 cells in the presence of FucA, Dex-Gal, and BLD4 (6.25 to 50 μM). C+: Cells treated with hydrogen peroxide (0.4 mM). C−: DMEM/SFB-treated cells. The symbol * indicates a statistical difference between the samples and the control (*p* < 0.05).

**Figure 8:**
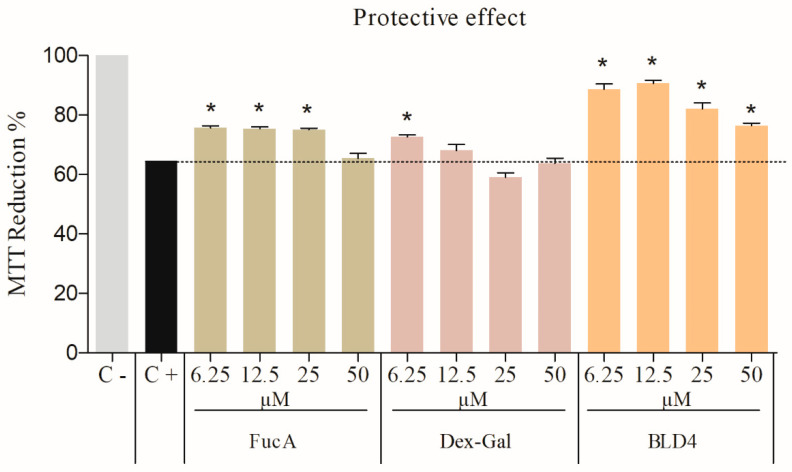
Oxidative stress induced by hydrogen peroxide—protective effect. The reduction of MTT to formazan by 3T3 cells in the presence of FucA, Dex-Gal, and BLD4 (6.25 to 50 μM). C+: Cells treated with hydrogen peroxide (0.4 mM). C−: DMEM/SFB-treated cells. The symbol * indicates a statistical difference between the samples and the control (*p* < 0.05).

With this in mind, the effects of BLD4, FucA, and Dex-GA on cells exposed to peroxide were evaluated. We categorized the evaluation into two types: concomitant action, where cells were exposed to peroxide and the samples simultaneously, and protective action, where cells were first exposed to the samples and then to peroxide (for details, see the Section 3). Therefore, the antioxidant effects of FucA, Dex-Gal, and BLD4 on cells were assessed using an assay with oxidative stress induced by H_2_O_2_. The results, depicted in Figure 7 and Figure 8, revealed intriguing findings.

Under the concomitant condition, where cells were simultaneously exposed to samples and H_2_O_2_, a notable difference of a 6% to 9% reduction in MTT compared to the positive control (cells exposed only to H_2_O_2_) was observed in the group of cells exposed to BLD4 (from 6.25 μM and 12.5 μM). Conversely, for the other samples, no significant reduction in MTT above the positive control was noted across all tested concentrations (Figure 7).

Figure 7 shows that only cells incubated with BLD4 were able to reduce MTT more efficiently than cells exposed solely to H_2_O_2_. This suggests that BLD4 was effective in mitigating the damage caused by H_2_O_2_. The formulation of the blend appears to enhance the antioxidant effect of the two polysaccharides against oxidative stress induced by peroxide.

This action of BLD4 could be attributed to at least two mechanisms. Firstly, it might involve the sequestration of peroxide, though this seems unlikely as neither component of BLD4 has demonstrated such activity [24,25]. Alternatively, BLD4 may induce cellular signaling pathways that enhance the cell’s own defense mechanisms against oxidative stress. This latter possibility is more plausible, considering that sulfated heteroglucan from the seaweed *Ulva pertusa* has been shown to prevent oxidative damage in human kidney cells (HEK-293) caused by H_2_O_2_. It achieved this by increasing the activity of intracellular enzymes, specifically superoxide dismutase (SOD) and catalase (CAT), at concentrations of 100 and 200 µg/mL for SOD and 200 µg/mL for CAT [48].

In the protective condition, wherein cells were pre-exposed to the test samples before H_2_O_2_ exposure, cells treated with FucA or BLD4 exhibited a remarkable reduction in MTT compared to the positive control. Specifically, cells exposed to FucA displayed a 75% reduction in MTT to formazan, while those treated with BLD4 exhibited percentages between 88 and 90% of MTT reduction, representing approximately 30% more than the positive control (Figure 8).

Li et al. [49] also obtained similar results. These authors prepared a mixture of polysaccharide derived from soybean seeds and vitamin E and assessed its ability to protect kidney cells from oxidative damage induced by peroxide. The blend proved to be significantly more effective as an antioxidant compared to its isolated components when evaluated at the same concentration.

Based on the data observed in Figure 8, the possibility that BLD4 is stimulating the expression of endogenous antioxidant agents cannot be ruled out. We plan to investigate this potential in future studies.

## 3. Materials and Methods

### 3.1. Materials

A total of 15 kDa dextran from *Leuconostoc mesenteroides*, nitro blue tetrazolium (NBT), toluidine blue, 1,3-diaminopropane, Coomassie brilliant blue R-250, 2,2′,2″,2‴-(Ethane-1,2-diyldinitrilo) tetra-acetic acid (EDTA), ascorbic acid, methionine, ammonium molybdate, gallic acid, L-ascorbic acid, and Dulbecco’s Modified Eagle Medium (DMEM) were all purchased from Sigma (St. Louis, MO, USA). Sterile fetal bovine serum—FBS—was purchased from Cultilab (Campinas, SP, Brazil). Penicillin and streptomycin were obtained from Thermo Fisher Scientific (Waltham, MA, USA). The 21 kDa fucoidan from *S. schröederi* used in this work was purified and characterized by Rodrigues-Souza et al. (2023) [19]. They promptly donated the fucoidan to us for this study. All other solvents and chemical products used in this study were of analytical grade purity.

### 3.2. Conjugation of Dextran with Gallic Acid (GA)

The conjugation of dextran with GA was performed according to the method described by Queiroz et al. [25]. A solution of 10 mg/mL dextran (15 kDa) was incubated for 30 min with 54 mg of ascorbic acid and 1 mL of hydrogen peroxide (1.0 M). After this period, GA was added at a ratio of 1 mol GA per mol of dextran repeating unit, considering a dextran disaccharide as the repeating unit. After a reaction period of 24 h, the solution was centrifuged using 3 kDa cut-off filters to remove free GA. The molecular weight of Dex-GA was determined by HPLC as described earlier [25] and was found to be 11.0 kDa.

### 3.3. The Determination of the GA Percentage

The percentage of GA present in Dex-Gal was determined using a colorimetric assay with the Folin–Ciocalteu reagent, as described by Queiroz et al. (2019) [25]. GA (Sigma) was used as the standard.

### 3.4. Fourier-Transform Infrared Spectroscopy (FT-IR)

FT-IR analyses were conducted using a Shimadzu FTIR-8400S spectrometer (Shimadzu Co., Nakagyo-ku, Kyoto, Japan). Samples of Dex and Dex-Gal were analyzed over 20 scans ranging from 400 cm^−1^ to 4000 cm^−1^.

### 3.5. Formulation of Blends Containing FucA and Dex-Gal Using Factorial Design

The formulation of the blends was based on a 2^2^ factorial design aimed at analyzing the influence of FucA and Dex-Gal concentrations on the response variables. These variables included antioxidant capacity, % hydroxyl radical scavenging, and % reducing power. The methodology followed was as per Padilha et al. [50].

In this design, the actual concentration values of FucA and Dex-Gal were coded as +1 and −1, representing levels above and below the midpoint, respectively. The midpoints (0) for the concentrations of both FucA and Dex-Gal were set at 50%. The lower points were established at 0% (−1) and the upper points at 100% (+1). A concentration of 0.5 mM was equated to 100% for both Dex-Gal and FucA. The coding of concentrations to levels is illustrated in Table 3 below.

After assigning codes to the levels of FucA and Dex-Gal concentrations, these levels were paired to create specific combinations used in the experimental blends. 

These pairings are detailed in the factorial planning matrix (Table 4), leading to the formulation of five unique combinations (Table 5). These combinations are designated as BLD1, BLD2, BLD3, BLD4, and BLD5. Notably, there is a duplicate at the central point to ensure robustness in the analysis.

Each sample (BLD1 to BLD5) varies in terms of FucA and Dex-Gal concentrations, allowing this study to assess the impact of these variations on the response variables such as antioxidant capacity, hydroxyl radical scavenging, and reducing power. The central duplicate (BLD5) serves to verify the consistency of the results at the midpoint concentration levels.

To prepare the samples, the polysaccharides (FucA and Dex-GA) were weighed and transferred into the same tube. Then, 1 mL of distilled water was added, and the tubes were shaken until the polysaccharides were fully dissolved and allowed to interact. The mass of each polysaccharide added was calculated to ensure that, after dissolution, their concentrations matched those specified by the factorial design, namely 0.25 mM and 0.5 mM. For BLD1, only distilled water was used.

### 3.6. In Vitro Antioxidant Tests

Three in vitro antioxidant tests were carried out to compare the antioxidant capacity of the formulated blends. Tests for total antioxidant capacity, reducing power, and hydroxyl radical (OH) scavenging were carried out as described by Presa et al. [51].

#### 3.6.1. Total Antioxidant Capacity (TAC) 

The assay was performed according to the method described by Presa et al. [51]. Samples were prepared in microtubes to which a solution containing ammonium molybdate (4 mM), sodium phosphate (28 mM), and sulfuric acid (0.6 M) was added. After incubation at 100 °C for 90 min, the microtubes were collected, and the absorbance was measured at 695 nm. Ascorbic acid (AA) was used as a standard, and the results were expressed as AA equivalents per gram of sample (Eq. AA/g).

#### 3.6.2. Reducing Power Assay

To evaluate the reducing power of blends, the samples were incubated for 20 min at 50 °C in a 0.2 M phosphate buffer (pH 6.6) which included 1% potassium ferricyanide. The reaction was halted by the addition of 10% trichloroacetic acid (TCA). Subsequently, 0.1% ferric chloride was introduced to the mixture. The absorbance was measured at 700 nm, with the results presented as percentages. In this assay, a standard concentration of ascorbic acid (0.1 mg/mL) was considered to represent 100% activity.

#### 3.6.3. Hydroxyl Radical Scavenging Activity Assay

In this assay, hydroxyl radicals were generated using a mixture containing 10 mM FeSO_4_·7H_2_O, 10 mM EDTA, 2 mM sodium salicylate, and 30% H_2_O_2_. A 150 mM phosphate buffer at pH 7.4 served as the negative control, while gallic acid was employed as the positive control. Test samples at a concentration of 10 mM were incubated with this reactive solution for one hour at 37 °C. The scavenging activity of hydroxyl radicals was then quantified using a microplate reader at a wavelength of 510 nm.

### 3.7. Antioxidant Tests on 3T3 Cell Lines

To assess the antioxidant effects of the blends on cells, cytotoxicity tests were initially performed on murine fibroblast (3T3) cells using the colorimetric MTT assay, which measures the reduction of MTT to formazan. Following this, tests for oxidative stress induced by hydrogen peroxide (H_2_O_2_) were conducted. FucA, Dex-Gal, and BLD4 were tested at concentrations of 6.25 μM, 12.5 μM, 25 μM, and 50 μM. In all tests, the cells were cultured under standard conditions (37 °C, 5% CO_2_) in Dulbecco’s Modified Eagle’s Medium (DMEM) supplemented with 10% fetal bovine serum (FBS) and antibiotics (penicillin and streptomycin).

#### 3.7.1. Assessment of Cellular Cytotoxicity

Cells were seeded in 96-well plates at a density of 5 × 10^3^ cells per well and cultured for 24 h under standard conditions (37 °C, 5% CO_2_) in Dulbecco’s Modified Eagle’s Medium (DMEM) supplemented with 10% fetal bovine serum (FBS) and antibiotics (penicillin and streptomycin). After 24 h, the culture medium was replaced with serum-free medium to induce cell starvation under the same conditions. The cells were then treated with media containing varying concentrations of FucA, Dex-Gal, or BLD4. Following the treatment period, the medium was removed, and MTT solution (1 mg/mL) was added to each well and incubated for 4 h. After this period, the MTT solution was removed and replaced with absolute ethanol to dissolve the formazan crystals. Absorbance was measured at 570 nm. The results were expressed as a percentage of MTT reduction, with the untreated control cells set as 100%.

#### 3.7.2. Assessment of Oxidative Stress Induced by Hydrogen Peroxide (H_2_O_2_)

This assay evaluates the capacity of the test samples to shield cells from damage induced by H_2_O_2_ under two different conditions: preventive and concomitant. The methodology for this assay was adapted from the procedures described by Fernandes-Negreiros et al. [35].

##### Determination of Oxidative Stress Condition

3T3 cells were exposed to varying concentrations of H_2_O_2_ to establish the optimal condition for inducing significant cell damage, approximately 50% cytotoxicity. Following the protocols outlined in Section 3.7.1, the cells were cultured, allowed to adhere, and then subjected to starvation. Subsequently, they were treated with H_2_O_2_ at concentrations ranging from 0.1 mM to 0.8 mM for 2 h. After this exposure, the medium containing H_2_O_2_ was removed, and the cells were incubated under standard culture conditions for an additional 24 h. Toxicity was then assessed using the MTT reduction assay described in Section 3.7.1. The concentration of H_2_O_2_ that resulted in 50% cellular toxicity was determined to be 0.4 mM, which was selected for use in subsequent tests.

##### Assessment of Concomitant Effect

This assessment involves exposing the cells to H_2_O_2_ concurrently with the test samples. Following the cultivation conditions previously described, the cells underwent a starvation period. After this, they were treated with 0.4 mM H_2_O_2_ along with FucA, Dex-Gal, and BLD4 for 2 h. Post-treatment, the medium containing H_2_O_2_ and the samples was removed, and the cells were then incubated under standard culture conditions for another 24 h. Subsequently, toxicity was evaluated using the MTT reduction assay, as detailed in Section 3.7.1. The positive control group was treated solely with 0.4 mM H_2_O_2_, while the negative control was treated with culture medium containing FBS. The results were expressed as a percentage reduction in MTT, with the positive control set as 100%.

##### Assessment of Preventive Effect

This assessment involves pre-treating the cells with test samples before inducing damage with H_2_O_2_. Following the same cultivation conditions as previously described, the cells underwent a starvation period. Subsequently, they were treated with FucA, Dex-Gal, and BLD4 for 24 h. After this pre-treatment period, the cells were exposed to a medium containing 0.4 mM H_2_O_2_ for 2 h, followed by another 24 h incubation under standard culture conditions. Toxicity was then evaluated using the MTT assay as detailed in item 3.5.1. The positive control was treated with 0.4 mM H_2_O_2_, while the negative control was treated with culture medium containing FBS. The results were expressed as a percentage reduction in MTT, with the positive control set as 100%.

### 3.8. Statistical Analyzes

Data are presented as the mean and respective standard deviations of at least three determinations made independently and in triplicate. Statistical analysis was performed using the ANOVA test, followed by the Bonferroni test (*p* < 0.05). All tests were carried out using Graphpad Prism 5.0 (2010) and the Statistica version 12.0 program 2013.

## 4. Conclusions

Through factorial planning and surface graph analysis, it was determined that the optimal blend formulation containing FucA and Dex-Gal for antioxidant testing was BLD4, which contains 100% of both components. BLD4 demonstrated an enhancement in the antioxidant activities of FucA and Dex-Gal when mixed in the ideal proportion. Additionally, BLD4 exhibited cytoprotective effects on 3T3 cells under oxidative stress induced by hydrogen peroxide. These findings suggest that the blend formulation of FucA and Dex-Gal possesses significant antioxidant potential and warrants further in-depth investigation for potential drug development.

## Figures and Tables

**Table 1:** Main bands found in FTIR spectrum in Dex and Dex-Gal.

Group	O-H	C-H	C=O (GA Ester)	C-C	C-O	α-(1→6)	C-O-C	α-D-Glucose
Dex	3394	2924	-	-	1461	1022	1129	869
Dex-Gal	3392	2927	1743	1543	1460	1020	1127	866

**Table 2:** Factorial design matrix with corresponding response variables.

	Response Variables		
Samples	TAC ^1^	Hydroxyl Radical Scavenging (%)	Reducing Power (%)
BLD1	0.0	0.0	0.0
BLD2	12.1	0.0	17.0
BLD3	16.7	26.0	12.0
BLD4	23.1	103.0	19.7
BLD5	12.7	31.0	14.0
BLD5.1	11.6	32.5	13.0

^1^ TAC is presented as equivalents of grams of ascorbic acid per gram of sample, as described in Section 3.

**Table 3:** Coding of concentrations to levels (2^2^).

Factor	Levels		
	(−1)	0	(+1)
FucA	0%	50%	100%
Dex-Gal	0%	50%	100%

−1 (low level) represents a concentration of 0%, indicating no inclusion of the factor in the blend; 0 (midpoint level) is set at 50%, serving as the baseline concentration for each factor; +1 (high level) corresponds to a concentration of 100%, representing the full inclusion of the factor in the blend.

**Table 4:** Factorial planning matrix.

Sample	FucA Level	Dex-Gal Level
BLD1	−1	−1
BLD2	+1	−1
BLD3	−1	+1
BLD4	+1	+1
BLD5	0	0
BLD5.1	0	0

**Table 5:** Blend composition.

	FucA		Dex-Gal	
Sample	Proportion	Concentration (mM)	Proportion	Concentration (mM)
BLD1	0.0	0.0	0.0	0.0
BLD2	100%	0.5	0.0	0.0
BLD3	0.0	0.0	100%	0.5
BLD4	100%	0.5	100%	0.5
BLD5	50%	0.25	50%	0.25
BLD5.1	50%	0.25	50%	0.25

## Data Availability

The original data presented in the study are included in the article, further inquiries can be directed to the corresponding author.
